# Metagenomic Investigation of Torque Teno Mini Virus-SH in Hematological Patients

**DOI:** 10.3389/fmicb.2019.01898

**Published:** 2019-09-18

**Authors:** Antonin Bal, Guy Oriol, Laurence Josset, Laurence Generenaz, Clémentine Sarkozy, Pierre Sesques, Gilles Salles, Florence Morfin, Bruno Lina, Jérémie Becker, Frédéric Reynier, François Mallet, Alexandre Pachot, Valérie Cheynet, Karen Brengel-Pesce, Sophie Trouillet-Assant

**Affiliations:** ^1^Laboratoire Commun de Recherche Hospices Civils de Lyon - bioMérieux, Centre Hospitalier Lyon Sud, Pierre-Bénite, France; ^2^Laboratoire de Virologie, Institut des Agents Infectieux, Groupement Hospitalier Nord, Hospices Civils de Lyon, Lyon, France; ^3^CIRI, Inserm U1111 CNRS UMR5308, Virpath, Univ Lyon, Université Lyon 1, Lyon, France; ^4^Service d’hématologie clinique, Centre Hospitalier Lyon Sud, Hospices Civils de Lyon, Pierre-Bénite, France; ^5^BIOASTER, Genomics and Transcriptomics Technological Unit, Lyon, France

**Keywords:** *Anelloviridae*, torque teno mini virus, metagenomics, hematological cancer, lymphoma

## Abstract

A new member of *Anelloviridae*, named torque teno mini virus (TTMV)-SH, was recently identified in the serum of three Hodgkin’s lymphoma patients suggesting that TTMV-SH may be associated with this type of hematological malignancy. We investigated by metagenomic analysis the presence of TTMV-SH-related viruses in plasma samples (*n* = 323) collected from patients with various hematological malignancies (multiple myeloma (MM, *n* = 256), non-Hodgkin’s lymphoma (NHL, *n* = 20), acute myeloid leukemia (*n* = 10)) and from healthy donors (*n* = 37). TTMV-SH-related strains were identified in 24 samples corresponding to four MM and one NHL patients. Phylogenic analysis revealed that the 24 isolates were close to the TTMV-SH strains previously identified, sharing 79.6–86.7% ORF1 nucleotide sequence identity. These results suggest that TTMV-SH-related viruses might be found in hematological diseases other than Hodgkin’s lymphoma. Due to the high genetic variability within *Anelloviridae* species, the association between a particular medical condition and a new genotype should be interpreted with caution.

We read with great interest the article of Pan et al. who identified a new member of *Anelloviridae* family, named torque teno mini virus (TTMV)-SH, in the serum of 3/19 Hodgkin’s lymphoma patients tested. This virus was not found in non-Hodgkin lymphoma nor in healthy donor samples, suggesting that TTMV-SH may be associated with this type of hematological malignancy (Pan et al., 2018).

## Objective of the Study and Clinical Samples Collection

We aimed to investigate the presence of TTMV-SH virus in a large collection of 286 plasma samples collected from patients (*n* = 72) with various hematological diseases as well as in 37 healthy donor samples. Seventy-two patients suffering from multiple myeloma (MM, *n* = 42), non-Hodgkin’s lymphoma (NHL, *n* = 20), or acute myeloid leukemia (AML, *n* = 10) were included in the cohort at diagnosis. In addition, 214 MM patient samples were collected prospectively up to 90 days after autologous stem-cell transplantation (ASCT, median follow-up: 255 days). Of note, MM and AML were not investigated in the study reported by Pan et al.

## Viral Metagenomics and Bioinformatical Analysis

Metagenomic analysis of plasma samples was performed using a validated workflow (Bal et al., 2018). Briefly, samples were centrifuged, filtered, and treated with DNase to reduce the human and bacterial components. Total nucleic acid was then extracted, randomly amplified, and prepared using Nextera XT DNA Library preparation kit according to the manufacturer’s recommendations (Illumina, San Diego, CA, USA). Libraries were sequenced on the Illumina Next Seq 500 platform with a median sequencing depth of 27,146,137 reads per sample. To retrieve potential sequences belonging to TTMV-SH virus, non-human NGS reads were first mapped on the complete genome of TTMV-SH published by Pan et al. (2018) using discontinuous megaBLAST. Selected reads were then aligned on the TTMV-SH ORF1 sequence with BBmap (version 36.32). The ORF1 consensus sequence was generated for each isolate and deposited in GenBank database under the accession numbers MN249910 to MN249933.

## Identification of Torque Teno Mini Virus-SH-Related Strains

**Figure 1:**
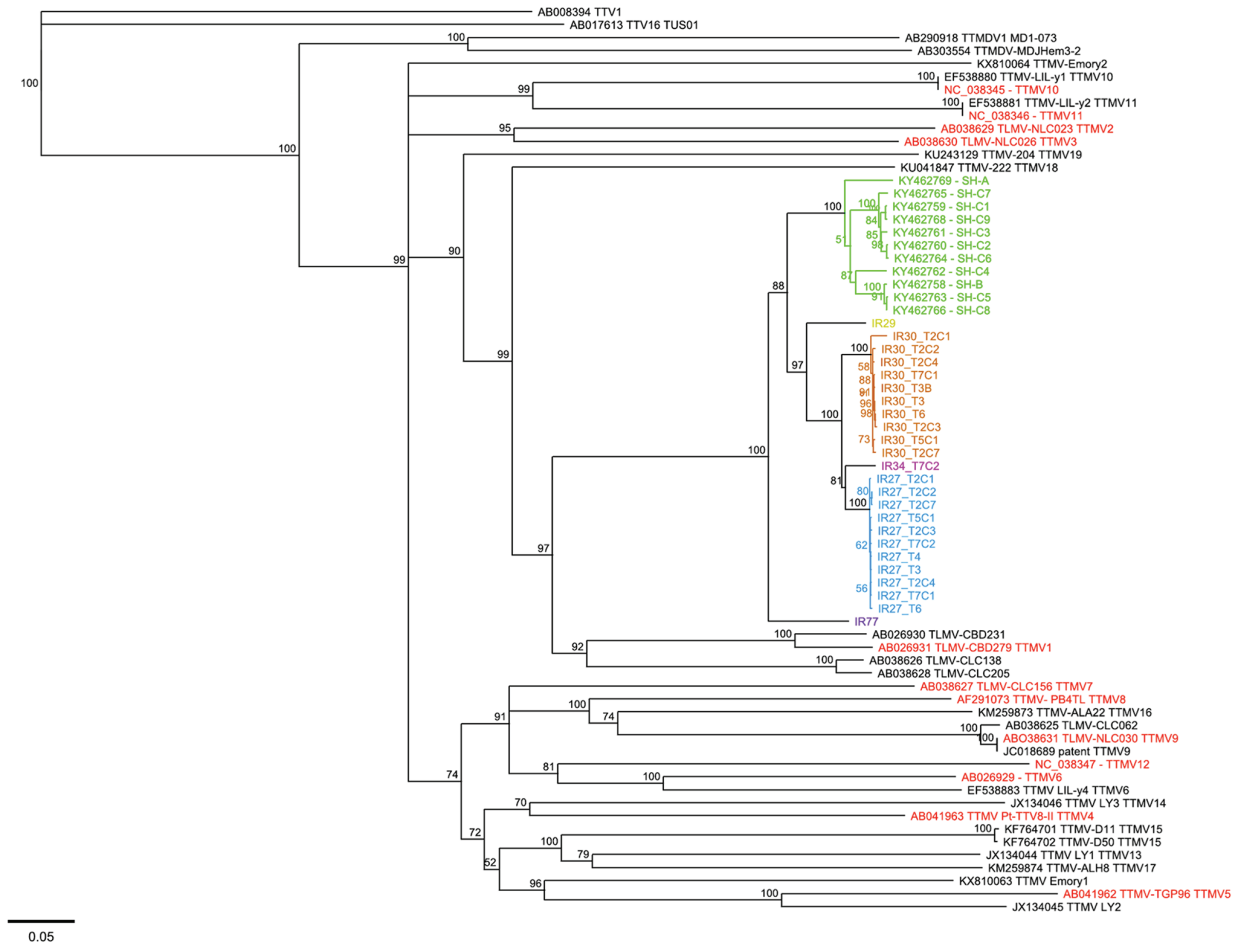
Phylogenetic analysis of TTMV strains based on the nucleotide sequence ORF1. TTMV sequences deposited in GenBank were downloaded. Four reference sequences of TTV and TTMDV strains were also used. Genetic distances were calculated with the Jukes-Cantor model of evolution. The tree was constructed by the neighbor-joining method using Geneious 10.0.7 software (Biomatters Ltd., Auckland, New Zealand) and validated using 1,000 bootstrap pseudo-replicates. Bootstrap values are indicated at each branching point. Scale bars indicate number of nucleotide substitutions per site. The green sequences indicate the 11 TTMV strains reported by Pan et al. (2018). The red sequences indicate the 12 TTMV species defined by ICTV. TTMV-SH-related sequences identified in the present study (*n* = 24) were represented by a different color for each patient (*n* = 5).

TTMV-SH-related strains, with the entire major coding region (ORF1) covered, were identified in 24 samples (median number of TTMV-SH-related reads detected: 52,189/sample). These 24 samples were collected from a total of five patients with MM (*n* = 4) and NHL (*n* = 1). For 2 MM patients, TTMV-SH-related viruses were identified in, respectively, 10 and 11 successive samples collected from diagnosis up to 90 days post-ASCT. Interestingly, TTMV-SH-related viruses were not detected in any of the 37 healthy donor samples tested. Phylogenic analysis revealed that the 24 isolates were genetically close to the TTMV-SH strains identified by Pan et al., sharing 79.6–86.7% ORF1 nucleotide sequence identity (i.e., sequence divergence ranging from 13.3 to 20.4%; Figure 1). According to the International Committee on Taxonomy of Viruses (ICTV), *Anelloviridae* species demarcation criteria are based on >35% cutoff value in nucleotide sequence divergence of the entire ORF1 [International Committee on Taxonomy of Viruses (ICTV), 2011]. Consequently, the results herein suggest that TTMV-SH-related viruses, belonging to the same cluster of species as Pan et al., might be found in hematological diseases other than Hodgkin’s lymphoma.

## Discussion

The complex relationship between the immune system and the viral replication cycle, as well as the high rate of recombination and mutation events, are responsible for a very high genetic variability within *Anelloviridae* species (Spandole et al., 2015). To date, 12 TTMV species have been approved by ICTV in the *betatorquevirus* genus [International Committee on Taxonomy of Viruses (ICTV), 2011] but we can hypothesize that additional species might be defined in the future.

The recent development of viral metagenomic approaches has allowed to enhance the characterization of *Anelloviridae* genetic diversity. Metagenomics contributed to identify new *Anelloviridae* genotypes in patients suffering from various diseases including Kawasaki disease, brain cancer, encephalitis, and periodontitis (Zhang et al., 2016; Ng et al., 2017; Thissen et al., 2018; Eibach et al., 2019). Due to their extreme genetic heterogeneity, it is possible that a new *Anelloviridae* strain could be characterized during particular medical conditions using metagenomic testing. Notably in case of compromised immune system, the *Anelloviridae* replication rate could be very high (Focosi et al., 2016) which may favor the emergence of a new genotype. However, the characterization of a new virus in patients suffering from a specific disease may not be sufficient to demonstrate an association and even less a causation.

As underlined by the study reported by Pan et al., the impact of viruses on cancer development should continue to be explored in larger cohorts even if the role of *Anelloviridae* may be difficult to establish. Furthermore, longitudinal studies are required to demonstrate a specific temporal association between a disease and a particular strain, as suggested by Koch’s postulates, revisited for molecular and metagenomics data (Falkow, 2004; Mokili et al., 2012).

## Ethics Statement

This non-interventional study received authorization from the French data protection body (*Commission Nationale de l’Informatique et des Libertés*—CNIL—agreement n_ DR-2015-694) and was approved by the national ethics committee (*Comité consultatif sur le traitement de l’information en matière de recherche*—CCTIRS, Paris, France—agreement n_15-529). All patients gave written informed consent.

## Author Contributions

AB, LJ, KB-P, FMo, BL, FMa, FR, AP and ST-A made substantial contributions to the conception and design of the study. CS, PS and GS are the guarantors for clinical data and sample collection. AB, LG and VC performed the sample preparations and sequencing. GO and JB performed bioinformatical analysis. All authors reviewed and approved the final version of the manuscript.

### Conflict of Interest Statement

GO, LG, FMa, AP, VC, and KB-P are employed by an in vitro diagnostic company, bioMérieux. JB and FR are employed by BIOASTER. AB has served as consultant to bioMérieux, and received a research grant from bioMérieux.

The remaining authors declare that the research was conducted in the absence of any commercial or financial relationships that could be construed as a potential conflict of interest.

## References

[ref1] Bal A. Pichon M. Picard C. Casalegno J. S. Valette M. Schuffenecker I. (2018). Quality control implementation for universal characterization of DNA and RNA viruses in clinical respiratory samples using single metagenomic next-generation sequencing workflow. BMC Infect. Dis. 18:537. 10.1186/s12879-018-3446-5 30373528 PMC6206636

[ref2] Eibach D. Hogan B. Sarpong N. Winter D. Struck N. S. Adu-Sarkodie Y.. (2019). Viral metagenomics revealed novel betatorquevirus species in pediatric inpatients with encephalitis/meningoencephalitis from Ghana. Sci. Rep. 9:2360. 10.1038/s41598-019-38975-z, PMID: 30787417 PMC6382885

[ref3] Falkow S. (2004). Molecular Koch’s postulates applied to bacterial pathogenicity – a personal recollection 15 years later. Nat. Rev. Microbiol. 2, 67–72. 10.1038/nrmicro799 15035010

[ref4] Focosi D. Antonelli G. Pistello M. Maggi F. (2016). Torquetenovirus: the human virome from bench to bedside. Clin. Microbiol. Infect. 22, 589–593. 10.1016/j.cmi.2016.04.007 27093875

[ref5] International Committee on Taxonomy of Viruses (ICTV) (2011). ICTV 9th report (2011). ssDNA viruses (2011). Available at: https://talk.ictvonline.org/ictv-reports/ictv_9th_report/ssdna-viruses-2011/

[ref6] Mokili J. L. Rohwer F. Dutilh B. E. (2012). Metagenomics and future perspectives in virus discovery. Curr. Opin. Virol. 2, 63–77. 10.1016/j.coviro.2011.12.004 22440968 PMC7102772

[ref7] Ng T. F. F. Dill J. A. Camus A. C. Delwart E. Van Meir E. G. (2017). Two new species of betatorqueviruses identified in a human melanoma that metastasized to the brain. Oncotarget 8, 105800–105808. 10.18632/oncotarget.22400 29285293 PMC5739680

[ref8] Pan S. Yu T. Wang Y. Lu R. Wang H. Xie Y. (2018). Identification of a torque teno mini virus (TTMV) in Hodgkin’s lymphoma patients. Front. Microbiol. 9:1680. 10.3389/fmicb.2018.01680 30093892 PMC6070622

[ref9] Spandole S. Cimponeriu D. Berca L. M. Mihăescu G. (2015). Human anelloviruses: an update of molecular, epidemiological and clinical aspects. Arch. Virol. 160, 893–908. 10.1007/s00705-015-2363-9, PMID: 25680568

[ref10] Thissen J. B. Isshiki M. Jaing C. Nagao Y. Lebron Aldea D. Allen J. E.. (2018). A novel variant of torque Teno virus 7 identified in patients with Kawasaki disease. PLoS One 13:e0209683. 10.1371/journal.pone.0209683, PMID: 30592753 PMC6310298

[ref11] Zhang Y. Li F. Shan T.-L. Deng X. Delwart E. Feng X.-P. (2016). A novel species of torque Teno mini virus (TTMV) in gingival tissue from chronic periodontitis patients. Sci. Rep. 6:26739. 10.1038/srep26739, PMID: 27221159 PMC4879676

